# Inhibitory effects of 405 nm irradiation on *Chlamydia trachomatis* growth and characterization of the ensuing inflammatory response in HeLa cells

**DOI:** 10.1186/1471-2180-12-176

**Published:** 2012-08-15

**Authors:** Cassandra J Wasson, Jessica L Zourelias, Nathan A Aardsma, Janis T Eells, Mike T Ganger, Justine M Schober, Troy A Skwor

**Affiliations:** 1 Biology Department, Gannon University, Erie, PA, 16541, USA; 2 Lake Erie College of Osteopathic Medicine, Erie, PA, 16509, USA; 3 Department of Biomedical Sciences, University of Wisconsin-Milwaukee, Milwaukee, WI, 53201, USA; 4 Department of Urology, University of Pittsburgh Medical Center Hamot, Erie, PA, 16507, USA; 5 Present address: Midwestern University, Downers Grove, IL, 60515, USA; 6 Present address: University of Buffalo, Buffalo, NY, 14260, USA; 7 Present address: Department of Pathology, University of Illinois at Chicago, Chicago, IL, 60612, USA; 8 Present address: Department of Chemical and Biological Sciences, Rockford College, 5050 E. State St, Rockford, IL, 61108, USA

**Keywords:** 405 nm, *Chlamydia trachomatis*, IL-6, CCL2, Phototherapy

## Abstract

**Background:**

*Chlamydia trachomatis* is an intracellular bacterium that resides in the conjunctival and reproductive tract mucosae and is responsible for an array of acute and chronic diseases. A percentage of these infections persist even after use of antibiotics, suggesting the need for alternative treatments. Previous studies have demonstrated anti-bacterial effects using different wavelengths of visible light at varying energy densities, though only against extracellular bacteria. We investigated the effects of visible light (405 and 670 nm) irradiation via light emitting diode (LEDs) on chlamydial growth in endocervical epithelial cells, HeLa, during active and penicillin-induced persistent infections. Furthermore, we analyzed the effect of this photo treatment on the ensuing secretion of IL-6 and CCL2, two pro-inflammatory cytokines that have previously been identified as immunopathologic components associated with trichiasis *in vivo.*

**Results:**

*C. trachomatis-*infected HeLa cells were treated with 405 or 670 nm irradiation at varying energy densities (0 – 20 J/cm^2^). Bacterial growth was assessed by quantitative real-time PCR analyzing the 16S: GAPDH ratio, while cell-free supernatants were examined for IL-6 and monocyte chemoattractant protein-1 (CCL2) production. Our results demonstrated a significant dose-dependent inhibitory effect on chlamydial growth during both active and persistent infections following 405 nm irradiation. Diminished bacterial load corresponded to lower IL-6 concentrations, but was not related to CCL2 levels. *In vitro* modeling of a persistent *C. trachomatis* infection induced by penicillin demonstrated significantly elevated IL-6 levels compared to *C. trachomatis* infection alone, though 405 nm irradiation had a minimal effect on this production.

**Conclusion:**

Together these results identify novel inhibitory effects of 405 nm violet light on the bacterial growth of intracellular bacterium *C. trachomatis in vitro*, which also coincides with diminished levels of the pro-inflammatory cytokine IL-6.

## Background

Trachoma continues to be the most common cause of preventable blindness worldwide. It has been estimated to visually impair between two and nine million people globally, although this may be an underestimate due to the lack of screening programs in endemic areas [1]. One of the etiologic agents is the obligate intracellular bacterium *Chlamydia trachomatis*[2], which is also the leading bacterial cause of sexually transmitted infections (STI) worldwide. These reproductive infections can lead to clinical symptoms such as urethritis, cervicitis, and pelvic inflammatory disease [3,4]. The ability of *C. trachomatis* to evade the immune system (reviewed in [5]) results in 70-90% of infected women and 30-50% of infected men being asymptomatic [6]. Due to repeated or persistent infections, or an absence of antibiotic treatment, ocular and reproductive tract sequelae can develop, resulting in corneal pacification and salpingitis respectively [4].

*C. trachomatis* has a unique biphasic life cycle involving both elementary and reticulate bodies. Elementary bodies (EBs) represent a metabolically inactive infectious phenotype capable of attaching to epithelial cells with subsequent internalization resulting in the formation of an inclusion body. Once inside the inclusion, the EB differentiates into a metabolically active reticulate body (RB) that multiplies via binary fission. As the inclusion grows, the RBs reorganize into EBs that are released from the host cell and can infect adjacent cells. These varying bioforms make treatment of chlamydial infections difficult. Furthermore, antibiotic therapies, exposure to IFNγ, or nutrient deprivation can lead to an atypical, persistent, non-cultivable, and morphologically aberrant intracellular state (reviewed in [7]).

Chlamydial infections in the conjunctiva and genitalia can incite an intense inflammatory response that, if chronic, can lead to scarring and fibrosis. Numerous pro-inflammatory cytokines, including TNFα, IL-1α, IL-6 and IL-18 [8,9], as well as a group of chemokines [8,10,11] responsible for the recruitment of leukocytes have been shown to be secreted from *C. trachomatis-*infected epithelial cells. This arsenal of cytokines and chemokines with incoming leukocytes results in the stimulation of both cellular- and humoral-mediated immune defenses. The type of host inflammatory response that is initiated with the infection determines the outcome of the infection. The current hypothesis is that resolution is mediated primarily by a dominant cell-mediated Th1 response, whereas chronic inflammation with subsequent scarring ensues if either the humoral Th2 response or regulatory T cells predominate (reviewed in [5]).

Development of chronic chlamydial infections is believed to arise as a result of immunopathology. This has been demonstrated by persistent elevation of pro-inflammatory cytokines like IL-6 among infertile women [12] and in tear fluid from post-scarring trachoma populations [13]. One study identified IL-6 secretion via the TLR2 signaling pathway after *C. trachomatis* infections [14]. This TLR2 pathway has been shown to be associated with fallopian-tube pathology, potentially contributing to the immunopathogenesis associated with *C. trachomatis* infection [14]. The chemokine monocyte chemoattractant protein-1 (CCL2) has also been identified in chronic chlamydial infections demonstrating elevated levels in post-scarring trachoma populations [13].

Due to the high prevalence of worldwide trachoma, the World Health Organization (WHO) established and supports the use of the SAFE (surgery, antibiotics, facial cleanliness, and environmental improvements) strategy to reduce disease transmission in endemic areas. Mass antibiotic therapy has been a mainstay in this program resulting in diminished prevalence of active chlamydial infections [15-17]. However, heightened recurrence rates of infection 6-24 months after termination of antibiotic therapy were evident in multiple studies [18-21]. Additionally, Burnham et al. saw an increase in chlamydia-associated STI Reinfection after a control program with antibiotic treatment was established [22]. The mass administration of antibiotics may lead to the development of antibiotic resistance in chlamydial species as well as other pathogenic bacteria. It is apparent that research into alternative treatments is warranted, and the use of phototherapy may be an attractive option.

Phototherapy utilizing low power lasers or light emitting diodes (LEDs) has been shown to reduce pain and chronic inflammation, and to promote tissue regeneration via a photochemical mechanisms (reviewed in [23]). Additionally, anti-bacterial effects due to the increased production of reactive oxygen species resulting in membrane instability and DNA damage have been evident with phototherapy [23-27]. Its use with several discrete wavelengths exhibits anti-bacterial activity requiring short treatment times without inducing anti-bacterial resistance subsequent to multiple treatment sessions [28].

In this study, we analyzed the effect of low-level 405 nm and 670 nm LED irradiation on the growth of *C. trachomatis* and the ensuing secretion of pro-inflammatory cytokines IL-6 and CCL2 from *C. trachomatis*-infected epithelial cells.

## Results

### Inhibition of chlamydial growth post - 405 nm irradiation

**Figure 1:**
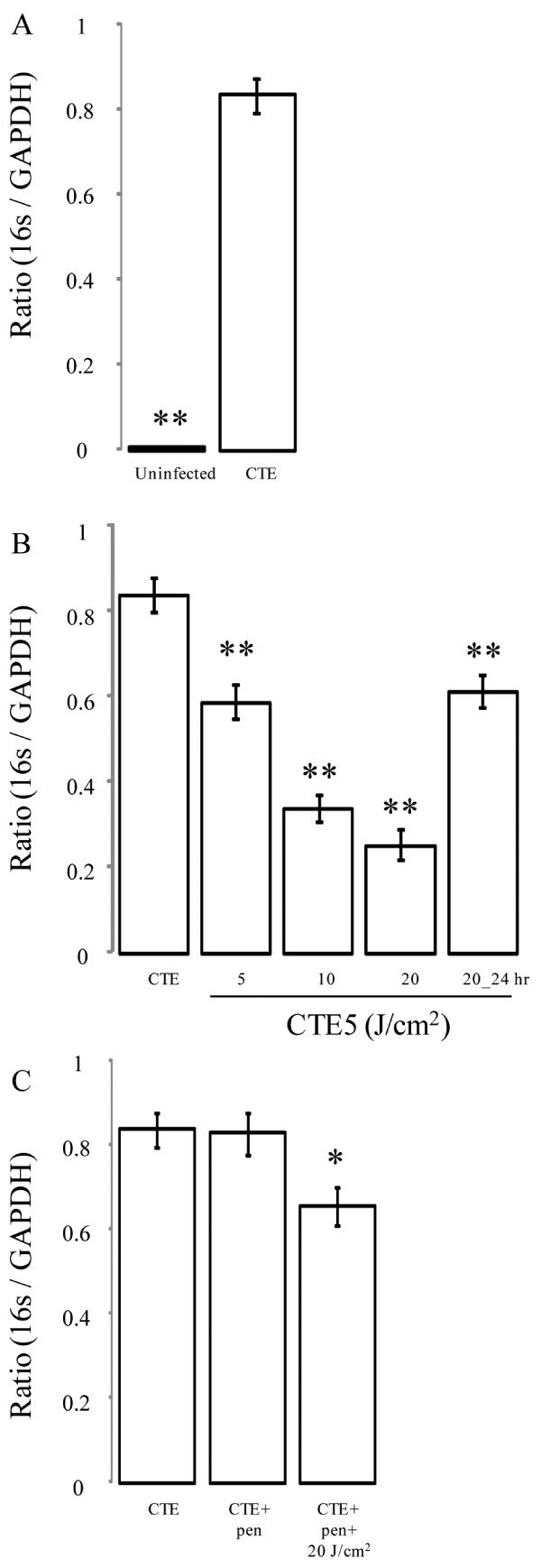
**Effects of 405 nm irradiance on chlamydial growth in HeLa cells.** (**A**) HeLa cells were infected with *C. trachomatis* serovar E at a MOI of 5. (**B**) Infected cells were then exposed to varying doses of 405 nm at a range of energy densities (5-20 J/cm^2^) either promptly after infection or 24 h post-infection (24 h post). Treatments are grouped based on post-hoc comparisons for convenience. The effect of 405 nm on chlamydial growth was assessed during active and persistent stages induced with penicillin (**B** and **C**). Growth was determined using quantitative real-time PCR to determine the ratio of chlamydial and eukaryotic housekeeping genes (16S: GAPDH respectively) 48 h post-infection on cDNA reverse transcribed from RNA. Mean ± standard deviation are plotted for the two replicated experiments. Statistical significance was determined post-hoc using a Bonferonni adjustment comparing all groups against *C. trachomatis*-infected HeLa cells alone (CTE); * *P* < 0.05, ** *P* < 0.005.

**Figure 2:**
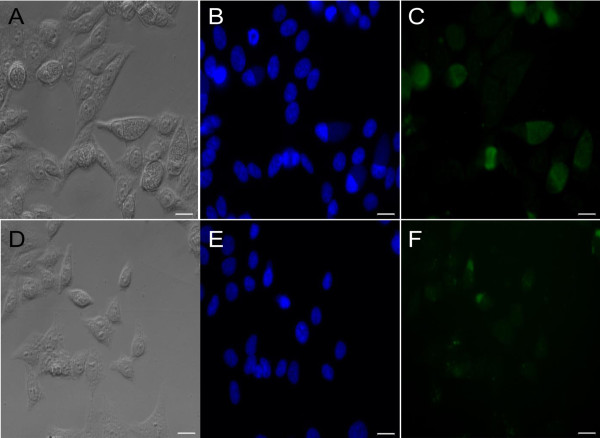
**Anti-chlamydial properties of 405 nm irradiance.** (**A**-**C**) HeLa cells were infected with *C. trachomatis* serovar E at a MOI of 5 without exposure to photodiodes. (**D**-**F**) Infected cells were exposed to 405 nm LEDs at 20 J/cm^2^ promptly after infection to evaluate anti-chlamydial effects during an acute chlamydial infection. Cells were fixed and stained with dapi (blue) (**B** and **E**) and anti-chlamydial (green) (**C** and **F**) antibody 48 hours post-infection. Bar = 10μm.

This study assessed the use of 405 nm and 670 nm LEDs as an alternative treatment against chlamydial infections. In Figure 1A, HeLa cells were infected with *C. trachomatis* at a multiplicity of infection (MOI) of 5. Irradiation treatment with violet 405 nm LEDs demonstrated chlamydial growth inhibition at energy densities as low as 5 J/cm^2^ (Figure 1B, *P* < 0.005). Anti-chlamydial properties were dose dependent with the strongest effect visible at 20 J/cm^2^ (Figure 1B, *P* < 0.005). Fluorescent staining (FITC) for chlamydial antigens supported an impairment of chlamydial growth and inclusion body expansion after 405 nm exposure (Figure 2D-F, 20 J/cm^2^) compared to *C. trachomatis* infected cells alone (Figure 2A-C). We also analyzed the effect of irradiation application time post-infection to determine if it was growth phase specific. At 24 h post-infection, irradiation with 405 nm (20 J/cm^2^) LEDs still demonstrated a significant growth inhibition (Figure 1B, *P* < 0.005). *C. trachomatis*-infected cells treated with red 670 nm LEDs at similar energy densities (5-20 J/cm^2^) showed no significant effect on growth (data not shown).

Considering many chronic chlamydial infections are in a persistent stage of growth, we tested the effect of 405 nm on chlamydial growth after penicillin-induced persistence. As shown in Figure 1C, 405 nm retarded chlamydial growth during a persistent state (*P* < 0.05) at 20 J/cm^2^, though the result was not as pronounced as it was in the active state. Once again, no effect was seen with 670 nm treatment (data not shown).

### The effect of 405 nm irradiation on IL-6 production in *C.* trachomatis-infected HeLa cells

**Figure 3:**
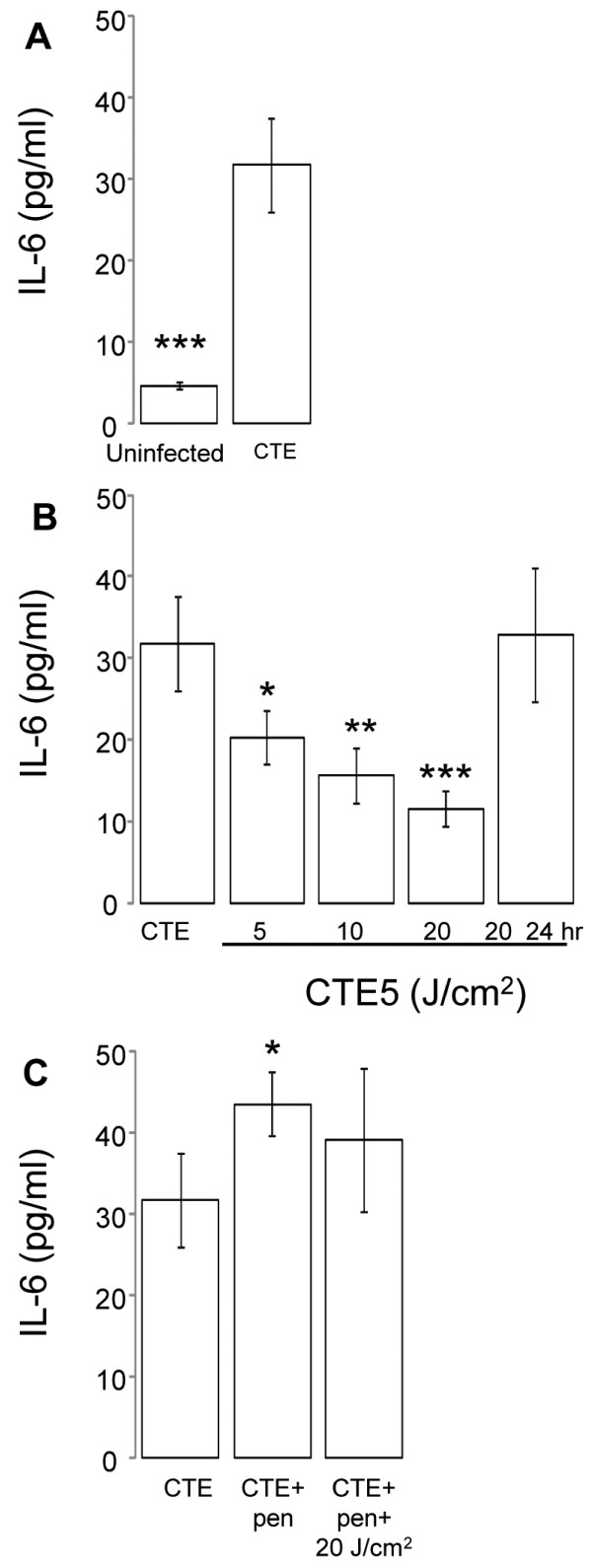
**Effect of 405 nm on IL-6 production in *C. trachomatis* -infected epithelial cells.** (**A**) HeLa cells were infected with *C. trachomatis* serovar E at a MOI of 5 (CTE5). (**B**) Infected cells were then exposed to varying doses of 405 nm at a range of energy densities (5-20 J/cm^2^) either promptly after infection or 24 h post-infection (post-24 h). The effect of 405 nm on IL-6 production was assessed during active (**A** and **B**) and penicillin-induced persistent stages (**C**). Supernatants were collected and measured for IL-6 production using an ELISA. Treatments are grouped based on post-hoc comparisons for convenience. Mean ± SEM are plotted for the two replicated experiments. Statistical differences were determined post-hoc using a Bonferonni adjustment comparing all groups to *C. trachomatis* infected cells (CTE); *, *P* < 0.05; ** *P* < 0.005.

Previous studies have identified IL-6 as a pro-inflammatory cytokine associated with immunopathologic effects in chronic *C. trachomatis* infections [12,13]. In this study, we demonstrated elevated IL-6 levels post-chlamydial infection compared to uninfected cells (Figure 3A, *P <* 0.005). *C. trachomatis* infected HeLa cells were treated with varying 405 nm energy densities (5-20 J/cm^2^) promptly after infection. Irradiation with 405 nm at energy densities of 5, 10, and 20 J/cm^2^ diminished IL-6 secretion in a dose-dependent manner 48 h post-*C. trachomatis* infection when compared to *C. trachomatis* infection alone (Figure 3B, *P* < 0.05, *P* < 0.05, and *P* < 0.005 respectively). Considering the potential for clinical therapies, we tested whether the effect of this phototherapy was dependent upon the 405 nm application time post-chlamydial infection. If applied 24 h post-infection rather than two hours, the significant 405 nm effect on IL-6 was lost (Figure 3B).

Due to the elevated levels of IL-6 with chlamydia-induced chronic grades of disease, we determined whether penicillin-induced persistence of a *C. trachomatis* infection *in vitro* would mimic the above clinical inflammatory signs. We demonstrated that persistence induction by penicillin significantly increased IL-6 production compared to *C. trachomatis* infection alone (Figure 3C, *P* < 0.05). The absence of IL-6 production above mock-infected levels from HeLa cells stimulated with 200 U/ml of penicillin alone indicates this effect was not cumulative (data not shown). No significant effects were evident on IL-6 production after 405 nm (Figure 3C) or 670 nm (data not shown) irradiation in this penicillin-induced persistent state.

### The effect of 405 nm irradiation on CCL2 production in *C. trachomatis* infected HeLa cells

**Figure 4:**
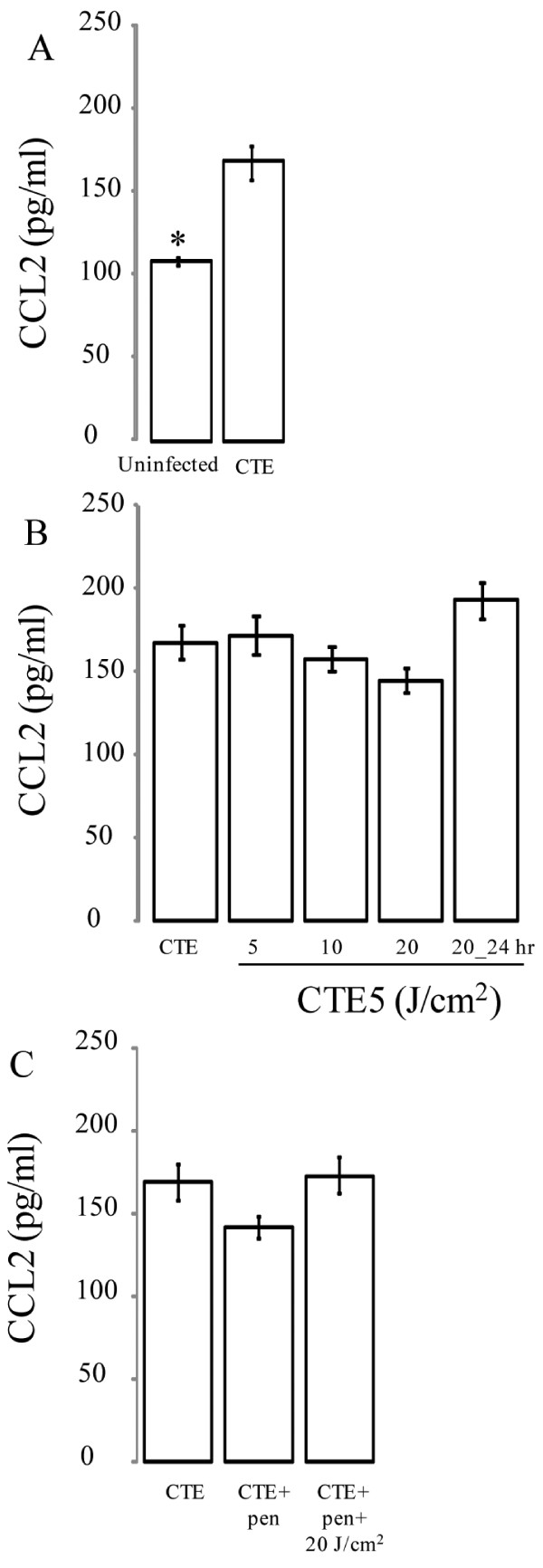
**Effect of 405 nm on CCL2 production in *C. trachomatis* -infected epithelial cells.** (**A**) HeLa cells were infected with *C. trachomatis* serovar E at a MOI of 5 (CTE5). (**B**) Infected cells were then exposed to varying doses of 405 nm at a range of energy densities (5-20 J/cm^2^) either promptly after infection or 24 h post-infection (post-24 h). The effect of 405 nm on CCL2 was assessed during active (**B**) and persistent stages induced with penicillin (**C**). Supernatants were collected and measured for CCL2 production using an ELISA. Treatments are grouped based on post-hoc comparisons for convenience. Mean ± SEM are plotted for the two replicated experiments. Statistical differences were determined post-hoc using a Bonferonni adjustment comparing all groups to *C. trachomatis* infected cells (CTE); *, *P* < 0.001.

Due to the involvement of CCL2 with acute and chronic grades of chlamydial infections [13,29] and its association with a Th2-mediated response [30], we evaluated the effect of 405 nm photo treatment on its production. In Figure 4*C. trachomatis* infection increased production of CCL2 in HeLa cells relative to uninfected cells (Figure 4A, *P* < 0.05). Though a diminishing pattern was evident for CCL2 production with increasing 405 nm energy densities (Figure 4B), 405 nm treatment failed to demonstrate any significant difference in CCL2 production compared to *C. trachomatis* infection alone. Unlike IL-6, penicillin-induced *C. trachomatis* persistence did not significantly influence CCL2 levels (Figure 4C) *in vitro*.

## Discussion

Multiple studies have demonstrated inadequate long-term protection of azithromycin for treating trachomatous trichiasis [19-21]. Suboptimal efficacy of antibiotics was also evident amongst a chlamydia-associated reactive arthritis population where persistent chlamydial bodies were identified in fibroblasts and macrophages one month after doxycycline treatment [31]. Further support for the poor antibiotic efficacy against chronic *C. trachomatis* infections was demonstrated in a population of women with post-infectious tubal infertility who remained infected despite antibiotic treatment [32]. Urogenital chlamydial re-infections have been identified as probable treatment failure with azithromycin or doxycycline using *ompA* genotyping in approximately 8 and 13.7% of cases [33,34]. Together the suboptimal efficacy of therapeutic antibiotics in the treatment of active and persistent chlamydial infections indicates the need for alternative treatments.

One potential alternative treatment utilizing 405 nm irradiation was evaluated in this study and demonstrated photo inactivation of *C. trachomatis* during active and persistent states. Small dosages starting at 5 J/cm^2^ had a significant growth inhibiting effect, with increasing energy densities positively correlating with growth inhibition. Therapeutic utility and clinical safety have been described using LED phototherapy at 405 nm against acne vulgaris [35] and gastric *Helicobacter pylori* infections, with the latter applied to the gastrointestinal mucosa via a light wand [36]. *In vitro* anti-bacterial activity of 405 nm irradiation has been demonstrated against multiple medically relevant Gram-positive and Gram-negative extracellular pathogens like *Staphylococcus aureus* (including methicillin-resistant strains, MRSA), *Streptococcus pyogenes, Pseudomonas aeruginosa, Clostridium perfringens, Campylobacter jejuni, Salmonella enteritidis* and *Escherichia coli*[24,37]. Overall Gram-negative bacteria appear more resistant to 405 nm irradiation than Gram-positive, with the exception of *Enterococcus faecalis.* The majority of Gram-positive bacteria appeared to require less than 11 J/cm^2^ for a log_10_ reduction in colony forming units, whereas Gram-negative bacteriocidal effects were apparent between 25 and 96 J/cm^2^[24]. The portable LEDs used in this study were battery operated with 88 second dosing times, therefore making it difficult to obtain higher energy densities. Comparing the log_10_ reduction levels of other Gram negative bacteria with *C. trachomatis* is difficult due to its intracellular nature and considering it may exist as two-uncultivable life cycle forms: RBs and aberrant persistent forms. After irradiation with an energy density of 20 J/cm^2^ we demonstrated a nearly 70% reduction in chlamydial growth, reflecting levels similar to other Gram-negative bacteria. To our knowledge, this is the first data demonstrating chlamydial growth inhibition caused by 405 nm irradiation.

Photo inactivation by 405 nm irradiation is believed to be caused by excitation of androgenic porphyrins, resulting in oxygen free radical production and subsequent bacterial membrane disruption [38]. Endogenously produced porphyrins, like coproporphyrin, uroporphyrins, and protoporphyrin IX, have been shown to be produced by both Gram positive and negative bacteria [25,39] though, to our knowledge, porphyrin production by *C. trachomatis* has not yet been demonstrated. Considering the intracellular nature of *C. trachomatis,* a second photo inactivation mechanism might be associated with altered expression of eukaryotic proteins in response to 405 nm irradiation. Boncompain et al. demonstrated a transient upregulation of reactive oxygen species within *C. trachomatis-*infected HeLa cells for approximately six hours after infection, with subsequent basal levels ensuing nine hours post-infection. The regulation of reactive oxygen species appears to be mediated by *C. trachomatis* sequestration of the NADPH oxidase subunit, Rac1, to the inclusion membrane [40]. Considering the significant growth inhibition effect when 405 nm was applied promptly two hours post-infection rather than 24 h, the irradiation might have altered chlamydial protein expression thus influencing its ability to sequester host Rac1, thereby increasing reactive oxygen species within the epithelial cells. An alteration in protein expression may have also delayed the formation and secretion of bacterial type III effector proteins, such as CPAF, that have previously been shown to be involved in binding and degrading eukaryotic proteins like cytokeratin 8, adhesion protein nectin-1, host transcription factor RFX5, and multiple host pro-apoptotic BH3 proteins [41-44]. Alternatively, the lack of 405 nm photo inactivation effect on chlamydial growth at 24 h post-infection might be due to the exponentially higher bacterial burdens within the inclusion body 24 h post-infection relative to two hours post-infection, potentially causing the differences after treatment to be less pronounced. It is also possible that multiple mechanisms co-exist and provide a cumulative anti-proliferative effect on *C. trachomatis,* though further studies are warranted.

The immunopathologic sequelae from conjunctival and genital chlamydial infections are likely mediated through the secretion of a group of pro-inflammatory cytokines. In trachoma, we demonstrated elevated levels of IL-6 during both acute and chronic grades of infection, with detectable chlamydial cases exhibiting more pronounced concentrations [13]. The role of IL-6 in immunopathologenesis was also evident in women with ectopic pregnancies [45] and positively correlated with antibody titers against *Chlamydophila pneumoniae* amongst atherosclerotic patients [46]. In an attempt to mimic chronic chlamydial infections, *Macaca nemestrina* fallopian tubes received repeated *C. trachomatis* infections, which resulted in fibrosis and elevated IL-6, IL-10, IL-2, and IFNγ levels [47]. In TLR2 -/- KO mice infected with mouse pneumonitis (MoPn), decreased fibrosis and inflammation with in oviducts and mesosalpinx correlated with abated IL-6 concentrations [14]. To determine the immunologic correlation of persistence *in vitro* with clinical presentation, we quantified IL-6 in penicillin-induced *C. trachomatis* persistent infections in HeLa cells. We demonstrated similar increases in IL-6 production in persistent infections compared to active infections *in vitro*. A previous study looked at persistent infections with *C. pneumoniae* in the presence of iron-depletion, IFNγ and penicillin, and demonstrated slightly diminished production of IL-6 after 24 h and 48 h [48]. However, multiple experimental differences between these studies, including the use of different chlamydial species, might provide an explanation for the differences in results. For example, Peters et al. added penicillin 30 min after infection, followed by daily media change. This is in contrast to our study which added penicillin 24 h post-infection without a daily media change. Wang et al. provided more molecular details of this persistent state, demonstrating attenuated production of secreted chlamydial proteins from ampicillin-induced persistence of *C. trachomatis* infected HeLa cells [49], suggesting that secreted type III effector proteins like CPAF [42], Tarp [50], CT311 [51], and CT795 [52] may be involved in regulating IL-6 levels. We are unaware of any other studies that examine inflammatory differences associated with penicillin-induced persistence. The elevation of IL-6 after penicillin-induced persistence supports the importance of this model in elucidating other inflammatory mediators that may be associated with chronic infections *in vivo.* Further research on molecular characterizations and their immunostimulatory properties is needed to understand this *in vitro* antibiotic-induced persistent model.

Considering the immunopathologic response to chronic chlamydial infections, we were interested in determining the role of 405 nm irradiation on cytokines previously associated with immunopathogenesis. In our study, irradiation with 405 nm had a dose-dependent effect on IL-6 production with 20 J/cm^2^ causing a 64% reduction. However, this effect was most likely associated with a decreased bacterial burden since previous studies demonstrated elevated IL-6 from UV-A (340-450 nm) exposed fibroblasts [53,54] and minimal effects of UV-A (1 J/cm^2^) treated keratinocytes on IL-6 production [55]. Interestingly, attenuation of IL-6 after 405 nm treatment was only evident if 405 nm irradiation was applied promptly after infection; the effect was lost if applied 24 h post-infection. We believe that at this later time point, multiple chlamydial proteins were already secreted by type III secretory pathways into the host cytoplasm and interacted with pattern recognition receptors (PRRs) resulting in IL-6 production.

Previously, we have identified CCL2 as a risk factor for trichiasis [13], and therefore analyzed the effect of 405 nm irradiation on *C. trachomatis* induced CCL2 production. To our knowledge, our findings are the first to demonstrate elevated levels of CCL2 after *C. trachomatis* infection in HeLa cells. *In vivo* analysis has shown elevated mRNA levels of CCL2 at two days post-infection with *C. trachomatis* mouse pneumonitis (MoPn) strain [29]. Unlike IL-6, the use of 405 nm phototherapy on *C. trachomatis* infected HeLa cells did not have a significant effect on CCL2 production. More studies are needed to further understand the relationship between *C. trachomatis* infection and CCL2 production resulting in these inflammatory differences.

## Conclusions

With increasing evidence to support persistent infections amongst a percentage of chlamydial infections post-antibiotic treatment [18-21,32-34], it is important to look for alternative treatments. In this study, we have provided the first *in vitro* evidence for anti-bacterial effects against an intracellular bacterium, *C. trachomatis,* using 405 nm irradiation administered by portable LEDs. The reduction in bacterial numbers and IL-6 concentrations, and the clinical safety of 405 nm irradiation, supports further studies evaluating its use as a phototherapy against chlamydial infections within the conjunctival and reproductive tract mucosae. The ability of photo treatment to penetrate mucosal tissue layers was demonstrated within the gastric mucosa against *Helicobacter pylori* using 408 nm light [36]. Together, these data provide a plausible alternative treatment against chlamydial infections and expands the anti-bacterial properties of 405 nm irradiation to include intracellular bacteria.

## Methods

### Cell line and bacterial stock

Human cervical adenocarcinoma cell line HeLa 229 (HeLa) and *C. trachomatis* serovar E were kindly provided by Dr. Deborah Dean (Children’s Hospital Oakland Research Institute, Oakland, CA) and were used following previous protocols [56,57]. HeLa cells were cultured and maintained in minimal essential medium (MEM; Sigma Aldrich Corp., St. Louis, MO) lacking phenol red and supplemented with 2 mM L – glutamine, 1 mM sodium pyruvate and 10% fetal bovine serum (C-MEM).

*Chlamydia trachomatis* serovar E reference strain was propagated in HeLa cells as previously described [56]. Elementary bodies (EB) were isolated after homogenization with subsequent gradient ultracentrifugation and resuspended in 0.25 M sucrose, 10 mM sodium phosphate and 5 mM L-glutamic acid (pH 7.2), and stored at -80°C. Determination of inclusion forming units (IFUs) was performed as previously described using fluorescent microscopy [57].

### Establishment of active and persistent *C. trachomatis* infections

HeLa cells were cultured at 1 x 10^5^ cells/ml in 6-well tissue culture plates and incubated for 20-24 h at 37°C + 5% CO_2_ prior to infection. Cells were then infected with *C. trachomatis* serovar E at a multiplicity of infection (MOI) of 5 (CTE5) in sucrose-phosphate-glutamate (SPG) buffer (220 mM sucrose, 3.8 mM KH_2_PO_4,_ 10 mM Na_2_HPO_4_, 5 mM glutamate, 10 μg/ml gentamicin [MP Biomedical], 100 μg/ml vancomycin [Across Organics, Morris Plains, NJ], and 25 U/ml nystatin [MP Biomedical] at pH 7.4) or mock-infected with SPG alone for two hours while on an orbital shaker. Media was then aspirated, washed, and replaced with C-MEM. Persistent infections were induced 24 h post-infection by the addition of 200 U/ml of penicillin G (Sigma Aldrich Corp.).

### Photo treatment of *C. trachomatis-*infected cells

405 nm and 670 nm were emitted from a WARP 10® LED (Quantum Devices, Inc., Barneveld, WI) with an irradiance of 60 mW/cm^2^ delivering 5 J/cm^2^ in an 88 second dosing time within a 10 cm^2^ area. Measurements were performed by a Gigahertz-Optic Integrate Sphere with a BTS256 - LED tester (Gigahertz-Optic, Turkenfeld, Germany) following LED standards set by the National Institute of Standards and Technology. *C. trachomatis*-infected cells were exposed to 0, 5, 10, or 20 J/cm^2^ of 405 nm or 670 nm LEDs as previously described [58] at 2 h or 24 h post-infection. Infected cells not exposed to 405 nm or 670 nm LED and uninfected cells mock infected with SPG alone were performed on separate plates to ensure no LED exposure.

### Quantification of IL-6 and CCL2

Supernatants were harvested at 48 h post-infection and centrifuged 16,000 x g in a micro centrifuge to remove all bacterial and cellular debris. Cell-free supernatants were frozen at -80°C until further analyzed. Undiluted supernatants were quantified for IL-6 and CCL2 using ELISA Ready-SET-Go® plates following manufacturer’s protocol (eBioscience, Inc., San Diego, CA). Standard curves were performed with seven two-fold serial dilutions (IL-6: 3.12 – 200 pg/ml; CCL2: 16.2 – 1000 pg/ml with the respective recombinant human IL-6 or CCL2) and used to determine sample concentrations.

### RNA isolation, reverse transcription and real-time PCR analysis

RNA was isolated from adherent cells, as well as the cell pellet from supernatants post-centrifugation as mentioned above, using the QIAshredder and RNeasy kit (Qiagen, Valencia, CA) following manufacturer’s instructions. RNA was then treated with DNase (Promega, Madison, WI) to digest any contaminating genomic DNA and reverse transcribed with script cDNA synthesis reagents (Bio-Rad, Hercules, CA). Negative controls were included that were not exposed to reverse transcriptase. SYBR® Green PCR Master Mix (Applied Bios stems, Carlsbad, CA) amplified the cDNA with the following real-time primers: GAPDH forward 5’ – AACAGCGACACCCACTCCTC – 3’, GAPDH reverse 5’ –CATACCAGGAAATGAGCTTGACAA– 3’, chlamydia 16 F 5’ – TCGAGAATCTTTCGCAATGG AC – 3’, and chlamydia 16R 5’ – CGCCCTTTACGCCCAATAAA – 3’ as previously described [59,60]. Arbitrary units were assigned using standard curves with five 1:3 serial dilutions for each target gene. Samples were reported as ratios of 16S: GAPDH.

### Immunocytochemistry and microscopy

*C. trachomatis*-infected HeLa cells with or without 405 nm were fixed with ice-cold methanol for 10 min. After aspiration, culture wells were washed with PBS and then stained with rabbit anti-*C. trachomatis* EBs (Virostat, Portland, ME) for 1 h. Wells were washed five times with PBS and counterstained with 4’, 6-diamidino-2’-phenylindole, dihydrochloride (Dapi; Thermo Scientific, Rockford, IL) for 10 min. Photos were obtained using the Olympus IX51 Fluorescent Microscope with differential interference contrast (DIC) filters.

### Statistical analysis

Due to different light intensities used for the 405 nm and 670 nm experiments, data were analyzed separately. In addition, both the replicated 405 nm and 670 nm experiments were repeated and therefore variation was partitioned between the separate experiments using a blocking factor [61]. Separate one-factor analyses of variance (ANOVA) were used to determine if 16S: GAPDH ratio, IL-6, and CCL2 production varied with treatment. For 405 nm treatments, post-hoc contrasts consisted of comparing *C. trachomatis* infected cells with uninfected cells and also examining *C. trachomatis-*infected cells exposed to different 405 nm densities (5-20 J/cm^2^). Additionally, penicillin-induced *C. trachomatis* infection was compared to *C. trachomatis* infected HeLa cells alone and penicillin-induced *C. trachomatis* infection with 405 nm treatment. The Bonferonni method (40) was used to establish a critical *P*- value.

## Abbreviations

LED, Light emitting diodes; EBs, Elementary bodies; RBs, Reticulate bodies; CCL-2, Monocyte chemoattractant protein-1; CTE5, Chlamydia trachomatis serovar E MOI 5; SPG, Sucrose-phosphate-glutamate buffer.

## Competing interests

The authors declare that they have no competing interests.

## Authors’ contributions

CJW and JLZ: performed the experiments, acquired, analyzed and interpreted the data, and drafted the manuscript. NAA and MTG: made substantial contributions to the conception and design of experiments, interpretation of results, and drafted and critically revised the manuscript. JTE and JMS: made substantial contributions to the conception and design of experiments, interpretation of results, and critically revised the manuscript. TAS: performed the experiments, acquired, analyzed and interpreted the data, drafted and critically revised the manuscript. All authors read and approved the final manuscript.
